# Dynamic random lasing in silica aerogel doped with rhodamine 6G

**DOI:** 10.1039/c8ra04561e

**Published:** 2018-08-21

**Authors:** Niklaus Ursus Wetter, Adriana Ramos de Miranda, Édison Pecoraro, Sidney José Lima Ribeiro, Ernesto Jimenez-Villar

**Affiliations:** Centro de Lasers e Aplicações, CNEN-IPEN/SP Av. Prof. Lineu Prestes 2242 São Paulo CEP 05508-000 Brazil nuwetter@ipen.br; Instituto de Química de Araraquara, Universidade Estadual Paulista Júlio de Mesquita Filho Rua Prof. Francisco Degni 55 Araraquara São Paulo CEP 14800-900 Brazil

## Abstract

Silica aerogel is a lightweight material, well known for its good mechanical and thermal characteristics, but its optical properties have received less attention, because it is weakly scattering. Here we present for the first time the lasing properties and their complex dynamics of silica aerogel doped with R6G. It is shown that the *Q* factors of the lasing modes determine the operation of the laser, being either resonant or ASE-lasing. For resonant lasing, the number of resonators is easily varied and the number of modes in a single resonator and their emission frequency can be dynamically adjusted, making this a truly versatile photonics material.

## Abstract 2

We demonstrate dynamically adjustable resonant lasing in silica aerogel, which makes this a truly versatile photonics material.

## Graphical abstract

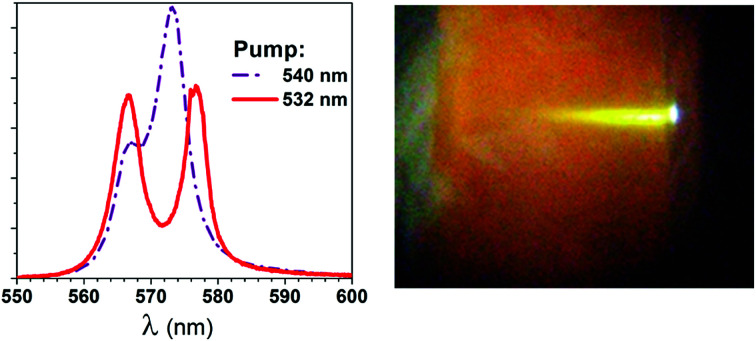

## 1. Introduction

The first model for the random laser (RL) was described by the diffusion equation, which disregards the wave nature and includes no phase information.^1,2^ Laser action is achieved by a significant increase in the length of the trajectory of light inside the volume where amplification occurs due to multiple scattering, increasing the gain length to an extent where it exceeds the loss length. This kind of feedback is devoid of any resonant characteristics of the electromagnetic field distribution inside the gain medium, *i.e.* the spatial distribution is independent of frequency and characterized by the absence of modes. Therefore, the laser frequency is determined only by the atomic transition, resulting in a single peak in the spectrum, which is the characteristic trait of lasers with non-resonant feedback or incoherent RLs.^3^ This single peak lasing is sometimes also referred to as amplified stimulated emission (ASE-lasing).

In 1994 Pradhan and Kumar predicted a qualitatively new phenomenon, called random lasing with coherent feedback, owing to the phenomenon of Anderson localization.^4–9^ In 1999 Cao and coworkers reported multiple sharp peaks emerging in a broad spectral range instead of one single band.^10^ The authors suggested that these sharp peaks are due to coherent feedback in the form of closed loop paths (localized states) by Anderson localization. Recently, similar narrow peaks have been reported in a liquid suspension random laser at the critical regime approaching localization.^11^ Much of the effort in explaining this phenomenon was invested in finding the resonators responsible for these coherent peaks. It has been suggested that random fluctuations of the refractive index can result in ring-shaped, macroscopically large resonators that are able to trap the light during long periods.^12^ Only the resonator with the highest *Q* factor stands out, which explains why generally only one resonator occurs within the pump spot. These light storage configurations are analogous to the so-called prelocalized states.^13^ Another alternative model for the RL was proposed in ref. 14, where the RL was treated as distributed feedback laser.^15^ This approach is similar to the resonator model above with the difference that, instead of dealing with ring-shaped resonators, the model assumes that there is a long range, almost periodic disturbance of the refractive index of Bragg type, which is responsible for the feedback loop.^16^

More recent developments in the field of RLs were due to a renewed attention to weakly scattering samples. Many narrow peaks were observed in the emission spectrum of samples with even very few scattering centers.^17^ The authors argued that the observed peaks do not require any kind of feedback and can be explained exclusively by assuming that some of the spontaneously emitted photons can travel distances much longer than on average (“lucky photon”) and thereby get strongly amplified. Later it was found that there are two types of peaks, the so-called spikes that can be detected without scattering centers and the peaks that need scattering centers.^18^ Spikes and peaks demonstrated significantly different statistical properties, which allowed the authors to attribute the origin of the first type to amplified spontaneous emission and the latter type to real lasing with coherent feedback. Even in the low scattering regime, characterized by strong radiative leakage as well as strong spectral and spatial overlap of the laser modes, individual modes (peaks) can still occur. This is only possible because, even in the weakly scattering system, there is a wide range of radiative lifetimes, which gain importance at the beginning of the laser action. The peaks of resonant feedback can be regularly spaced and the number of peaks depends on the geometry of the pumping volume.^19^

A major advantage of RLs over regular lasers is that their production is cheap, the required technology relatively simple and it is possible to produce RLs with several different materials like semiconductor nanoparticles,^10^ ceramic powders and polymers,^20,21^ organic materials,^22^ inorganic materials with chromophores,^23,24^ and biological tissues.^25,26^ Potential applications are speckle-free imaging in biology, remote-sensing, display technology, encrypting, cancer detection and distributed amplification.^25,27^ In order for their inherent advantages to become attractive for these applications, optimization is required principally with respect to laser efficiency. In order to overcome the problems of RLs associated to non-directional output and lack of efficiency, the main approach has been to choose low-dimensional RLs such as 1-D fiber and 2-D DFB random lasers that have achieved up to several watts of continuous output.^28,29^ However, the sophisticated production methods and sheer size of these devices is in stark contrast to what could be achieved with 3D RLs. For example, a 3D RL with 68 mrad beam divergence and another RL with 50% optical efficiency were demonstrated.^30,31^ Some applications such as wavelength-division multiplexing, optical sensing of single or multiple species and terahertz generation would benefit from stable, tunable, dual or even triple wavelength laser emission.^32,33^

As pointed out above, the appearance of equidistant thin peaks has been observed in weakly scattering media. In this work, we analyze the resonant modes of rhodamine 6G (R6G) in silica aerogel and demonstrate that they can be attributed to the modes of Fabry–Perot like resonators, which are delimited by the pumping depth. By choosing the size and shape of the pump volume, the number of resonant modes can be limited. In this way, the number of modes of a single resonator can be adjusted by applying different pump powers or pump frequencies, which in turn modifies the resonator size (pumping depth). Additionally, the pumping area also limits the number of lasing modes.

Silica aerogel (SA) is a material well known for its good mechanical and thermal properties. However, its optical properties have received less attention, because it is optically not transparent.^34^ For the same reason, low-density silica aerogel (LDSA) is an interesting host for random laser systems. For some applications, especially for space-based ones, it would be advantageous to combine the favorable characteristics of random lasers, pointed out above, with a stable and light matrix. SA has a high nonlinear optical coefficient (similar to that of glass) and negligible optical nonlinear absorption, which, amongst other applications, makes this material interesting as optical limiter and for high power lasers.^35^ The nonlinear index is increased by two orders of magnitude when rhodamine is introduced into the matrix, making this a material of impressive optical properties. Additionally, the silica inertness, which has allowed numerous applications,^36–39^ avoids greatly the R6G photo-degradation.^23,24,40^ In yet another application, silica aerogel was introduced into a hollow-core fiber where its low linear index of refraction and its material compatibility permitted wave guiding.^41^ The vast range of applications of RLs and the favorable material properties of SA, combined with its interesting optical properties when doped with rhodamine, make it a promising material for photonic devices.

## 2. Methods and optical characterization

**Fig. 1:**
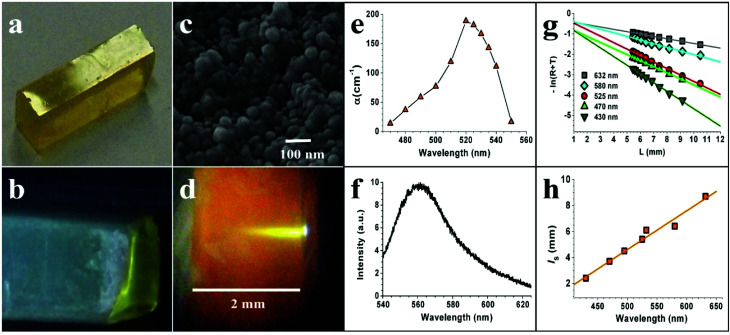
(a) Undoped aerogel slab when illuminated from the top. (b) Undoped aerogel slab when illuminated with white light from the left end. (c) SEM image of the silica aerogel samples doped with R6G. (d) Side view of a sample of 2 mm width and impinging laser beam. (e) Measured absorption coefficient as a function of wavelength. (f) Fluorescence emission spectra for a pump wavelength of 532 nm. (g) Measurement of −ln(*R* + *T*) as a function of sample thickness *L* for several incident wavelengths achieved by rotating the undoped sample. (h) Scattering mean free path as a function of wavelength (the orange linear fit is just a guide to the eye).

The reagents used for the synthesis of R6G doped silica aerogel were (acquired from Sigma-Aldrich) tetraethyl orthosilicate (TEOS 98%), hydrochloric acid (HCl 37%), ammonium hydroxide (NH_3_ 30%) and R6G (C_28_H_31_N_2_O_3_Cl 95%). Two step catalyzed TEOS based doped xerogels (hydrolysis and gelation) were prepared from ethanol (C_2_H_6_O), HCl and NH_4_OH. Doping with R6G was performed during hydrolysis. Before the gelation occurred, the sol was transferred to polystyrene molds (1 × 1 × 4 cm), which were kept covered at room temperature for 14 days of aging. After that, the slab-like monoliths were transferred into a CO_2_ critical point drier (Quorum Technologies, UK) and subjected to heat treatment to remove any residual solvent. The finished sample, with approximate dimensions of 5 × 5 × 20 mm (shown in Fig. 1a) and weight ratio of (R6G/SiO_2_) = 0.002,^42^ is composed of a single silica phase of amorphous and fractal structure, which acts as a scattering medium for light.^43,44^ Scattering effects can be observed with the undoped samples that have a foggy, semi-transparent appearance but when the material is illuminated by white light on dark background, a bluish reflection (Rayleigh scattering) and reddish transmission is clearly seen (Fig. 1b). Approximately 97% of the samples' volume is composed of pores filled with air. The estimated surface area of the material is 900–1200 m^2^ g^−1^. The size of the pores (*d*_SC_) that comprise the scattering structure (hollow silica shells filled with air) range from below 100 nm (Fig. 1c) to around 400 nm. However, the volume fraction occupied by these pores may vary notably in dimensions around some microns, *i.e.* the silica aerogel structure is inhomogeneous at dimension of microns, which is well known.^45^ In general, two different types of structures can be simultaneously found within the network. These are either “branches” or “pearl-necklaces” (which are just rotated branches so that they form closed loops). From the SEM images, it is inferred that the morphology of our samples is dominated by the latter structure.

Absorption measurements were carried out with the R6G doped silica aerogel matrix. It is assumed that the rhodamine molecules are attached to the walls of the silica and also embedded in defects such as cracks, which experimentally turned the samples very brittle.^46^ We remark that [R6G] varies notably inside the aerogel matrix, *i.e.* a strong inhomogeneity in [R6G] is expected, which has been previously reported for other dopants in the silica aerogel.^46^ Unlike the undoped sample of Fig. 1a, the doped sample broke up into pieces with dimensions of typically several millimeters as shown in Fig. 1d. Some pieces had complex shape and were inadequate for experiment. Three samples were chosen because they had two large, close to parallel surfaces of almost mirror-like appearance as if they were cleaved. These surfaces of 5 to 10 mm^2^ area were probed by the laser pump beam during our laser experiments. For the absorption measurements, the laser beam was attenuated to 28 μJ and the OPO's wavelength was varied from 470 nm to 550 nm in steps of 5 nm. The beam was focused close to the upper border of the sample with a *f* = 5 cm lens (fluence of 0.5 mJ mm^−2^) and an image was taken with a digital microscope through a 550 nm cut-off filter in order to record only the pump beam absorption (see Fig. 1d). The image of the absorbed laser beam was fitted along the optical axis with a single exponential function after having carefully avoided any camera saturation. Changing the position of the focus on the sample caused small changes of the absorption length after lateral translation of a couple of millimeters. A larger variation of up to ∼20% was measured for the same pump power using different samples. The [R6G] inhomogeneity inside the silica aerogel matrix may provoke a significant variation of the effective absorption length. Notice that a local [R6G] increase should give rise to a decrease of the effective absorption length (macroscopic absorption length), *i.e.* a decrease of the pumping depth. We remark that absorption measurements were performed at pumping powers well below RL threshold. Additionally, a decrease of R6G absorption is expected above RL threshold, which has been observed in previous works.^11,23^

The result of the measured absorption coefficient as function of wavelength is seen in Fig. 1e. The spectrum shows very close resemblance to measurements with R6G when only monomers are measured.^47^ Even details such as the shoulder appearing in the spectra at 500 nm are in agreement, which shows the relative accuracy of this absorption measurement.^47^ A peak absorption coefficient of 188 cm^−1^ was measured at 520 nm, corresponding to an absorption length (1/*α*) of *l*_*α*_ = 53 μm.

Fluorescence emission was measured with the pump beam just grazing the upper boarder of the sample in order to avoid any reabsorption and is shown in Fig. 1f. Peak emission was measured at ∼561 nm and emission bandwidth at FWHM was 37 nm. The wavelength of the peak emission is slightly lower than customary reported for R6G in ethanol solutions (563–564 nm) with TiO_2_ and/or TiO_2_@silica NPs as scattering particles.^23,24^ This could be due to the [R6G] being located on the silica–air interface of the aerogel, which could lightly change the energy bands of R6G due to the electric field near the silica surface.^48^

The scattering mean free path (*l*_s_) was measured as a function of wavelength by collecting ballistic photons as a function of sample thickness, assuming negligible absorption of our undoped sample.^49^ The pump source was a tunable OPO that supplied 9 ns pulses at 532 nm of up to 14 mJ of pulse energy with 20 Hz in a beam of 1.5 mm radius and quality factor of *M*^2^ = 11 in the horizontal direction and *M*^2^ = 16 in the vertical direction. For the measurements, the laser beam was attenuated to 1.7 mJ and then directed onto one of the larger side facets of the polished sample of Fig. 1a, which was positioned on a rotation stage. Upon rotating the sample away from normal incidence, it was possible to achieve a continuously variable physical path length (sample thickness), *L*, for the laser beam inside the sample, ranging from 5 mm to 11 mm length. At each rotation position the pulse energy reflected (*R*) from the first surface and the pulse energy transmitted (*T*) through the sample were measured by identical, calibrated detectors (Fig. 1g). If elastic scattering is the only loss mechanism, *l*_s_ is determined by fitting with *l*_s_ = −*L*/[ln(*R*(*L*) + *T*(*L*))].

As expected from the transparent appearance of aerogel, very long mean free paths are obtained as a function of wavelength, as shown in Fig. 1h, which categorizes this material as a weak scatterer. The same set-up described above was used to measure the undoped aerogel slab's minimal reflection for TM polarized light at the Brewster angle. From this Brewster angle a refractive index of approximately 1.17 (±0.08) was obtained for the undoped test sample.^50^

Coherent emission from the random laser, which is detailed in the next section, was measured by the same set-up described above. The pump beam passed through a dielectric beam splitter, inserted before the focusing lens, which then captured the signal backscattered from the sample and redirected it to an optical fiber port for spectral and temporal analysis.^19^ Spectral resolution was 0.06 nm. The focal length of the focusing lens was 10 cm and the samples were inserted 2 cm before the focus. Average beam radius at the sample position was *w*_1_ = 470 μm and maximum pump power was 8 mJ (fluence of 12 mJ mm^−2^). All acquisitions were made in single shot mode measuring simultaneously pump pulse energy, spectral signature and temporal behaviour for each shot.

## 3. Random laser characterization

Aerogel showed to be a rich source of different mechanisms for the generation of random lasing in low-scattering media. We found that by increasing the laser pump power on the aerogel it is possible to see, depending on the location of laser incidence, both narrowing of a single peak as well as the emergence of several peaks. These peaks can be separated spectrally in an irregular and chaotic fashion and change from pulse to pulse or, depending on location and pump intensity, they can also be separated by regular intervals and remain extremely stable for long periods of time and without changes from pulse to pulse. Moreover, when the peaks are regular, the separation between them can range from a fraction of a nanometer to more than 10 nanometers.

**Fig. 2:**
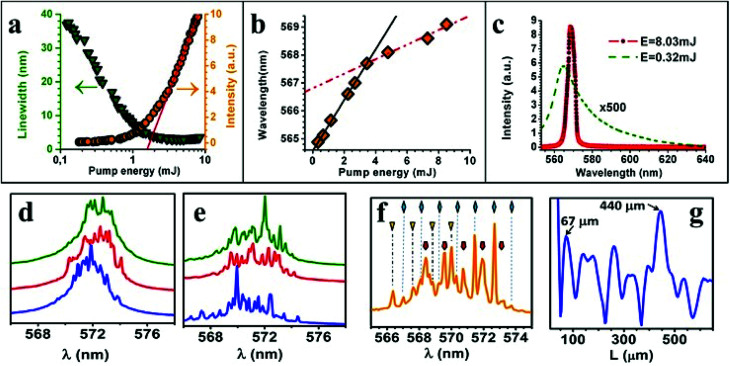
(a) Linewidth narrowing and output intensity as a function of input pump pulse energy at 532 nm. The red line's intersection with the *x*-axis determines the threshold energy of 1.7 mJ. (b) Red shift as a function of pump power. (c) Emission spectra for 8 mJ and 0.3 mJ of pump power (not to scale). Behaviour at threshold: (d) three consecutive spectra taken at low absorption (pump wavelength of 495 nm) and (e) high absorption (pump wavelength 515 nm). (f) Emission spectrum under 525 nm pumping showing three combs of equidistant peaks and (g) corresponding PFT spectrum showing an equivalent linear cavity length of 67 μm. The highest peak at 440 μm corresponds to the average spacing between peaks.

When probing the aerogel samples at high pump powers, the appearance was mostly of one, relatively broad, single amplified spontaneous emission (ASE) peak, as in ref. 3, in which case its characterization was made through analysis of the emission linewidth and laser intensity as a function of pump pulse energy, as shown in Fig. 2a. The intensity emitted as a function of pumping energy shows the two-phase input–output curve, typical of a laser. The threshold energy was estimated by the intersection of the in–out curve with the horizontal axis and was *E*_th_ = 1.70 mJ.

A red shift of the emission peak was observed as a function of pump power (Fig. 2b). This red shift is due to reabsorption and is a function of the population inversion in this three-level system.^51^ At higher pump intensity, the inversion decreases because higher ground-state sub-levels become occupied and, therefore, reabsorption matters, which explains the red shift increase. Maximum inversion is achieved with the onset of the linear slope in Fig. 2a at approximately 3 mJ. At still higher pump energies the inversion is clamped to its maximum value and the red shift changes less as shown by the dotted slope in Fig. 2b. Clear experimental evidences of above phenomenon has been reported in ref. 11 and 23, where a decrease of R6G absorption was observed above RL threshold. This latter gives rise to an increase of the depth of the pumping region, which leads to an increase of the effective photon path length (deeper gain region).

Although well above threshold we observed a single peak as a common feature in the vast majority of the probed sites, the behavior at threshold was different. Below threshold, an increase in pump energy would frequently start with a narrowing of the emission band followed by the occurrence of peaks. The peaks would appear on a more or less pronounced background (ASE) depending on the effective absorption length, *i.e.* the depth of the pumped region. Note that an increase of pumping depth (low absorption) must lead to an increase of the *Q* factor mainly for the ASE modes and, consequently, ASE modes are promoted. In Fig. 2d and e spectra of three consecutive pulses are shown for low absorption (495 nm pumping) and high absorption (525 nm pumping) obtained for 3 mJ of pump pulse energy (fluence of 4 mJ mm^−2^), respectively. It can be clearly seen that the incoherent background (ASE) is smaller at higher absorption (shallower pumping depth), which would mean a comparably lower *Q* factor for ASE modes promoting preferably the peak modes (Fig. 2e), that the frequency spacing is more regular and that more peaks do appear. The width of the peaks was 0.2 nm.

The gain could be adjusted in our experiment either by adjusting the pump power or pump intensity by choosing different focal lengths. At low absorption (deeper pumping region) the peaks would occur at random locations in the emission spectra although their frequency spacing would show regularity from pulse to pulse which could also be observed in the Fourier transform spectra (Fig. 2g). With more absorption (shallower pumping depth), the peaks would occur increasingly (ASE decreasing) at the same location and their frequency spacing would become very stable from pulse to pulse (Fig. 2e). This latter may be explained by a decrease of the ASE *Q*-factor due to the decrease of pumping depth.

We also calculated the PFT spectra of each pulse. In order to obtain a meaningful result from the FFT, we first transformed the emission spectra into *k*-space (wavenumber) and then multiplied the final result with 2π/*n*. As a result, we obtain a length scale, which can be compared to an equivalent linear laser cavity, a Fabry–Perot resonator. An example of an emission spectra and its corresponding PFT is seen in Fig. 2f and g respectively. Fig. 2f was obtained when pumping at the absorption peak of 525 nm with 3 mJ (slightly above the RL threshold). Three sets of peaks separated by a free spectral range (FSR) of 1.06 nm, 1.06 nm and 1.16 nm, respectively, can be identified. Average frequency spacing between adjacent peaks of different combs is 0.32 nm, which corresponds to the peak at 440 μm in the PFT spectrum of Fig. 2g. For higher pump absorption, a larger separation between peaks in the emission spectrum and a shorter cavity length in the PFT spectrum are observed. For comparison, the first peak in the PFT spectrum for low pump absorption (Fig. 2d) occurs on average at a cavity length of 180 μm and for higher pump absorption (Fig. 2e) at 100 μm. These Fabry–Perot cavity lengths obtained from the PFT spectra agree with the pump absorption lengths measured in Fig. 1e. The ratio between absorption length and cavity length is 0.8 in all three examples (Fig. 2d–f), indicating that the cavity length is determined by the length (depth) of the pumped region.

Such behavior has been observed prior in ref. 19. When regarding the random laser as a randomly distributed feedback laser, it can be treated similar to a traditional DFB (distributed feedback) laser operating in the under coupling regime.^52^ In this regime, the feedback is formed mainly by the scatterers near the borders of the pumped volume, which is defined by the pump spot size (*w*_1_) and the absorption depth (*l*_*α*_). This can be intuitively understood because outside this border exists strong reabsorption and inside this border, the modes with the highest *Q*-factor (gain) are those with the longest cavity length. Additionally, a highly directional emission of the resonator occurs along its axis, which makes the detection of resonators that lie in the plane of the pump spot much more difficult.

Given that *w*_1_ ≫ *l*_*α*_ we can expect several resonators to occur within the pump spot size, for pumping powers around the threshold, which accounts for the different frequency combs detected in Fig. 2f. To further investigate the dynamics of the peaks as a function of pump power, we reduced the number of combs to one by focusing tighter with an *f* = 3 cm focusing lens as opposed to the *f* = 10 cm focusing lens used before and moved the sample into the focus position (calculated beam radius of *w*_2_ = 80 μm). Pump wavelength was 532 nm. Note that a smaller pumping area should lead to a decrease of the number of modes of Fabry–Perot cavity until eventually only one mode is able to oscillate.

**Fig. 3:**
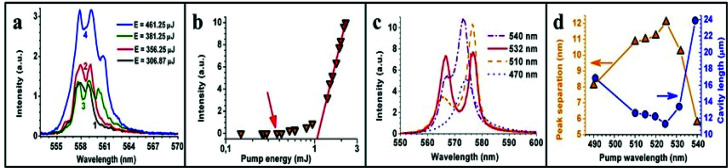
Below threshold behaviour: (a) pulse emission spectra at four different pump energies (wavelength of 532 nm) below threshold and (b) input–output measurements for the same pump spot. The intersection of the straight line with the *x*-axis indicates a threshold pump energy of 1.05 mJ. The arrow indicates the pump power region where the peaks appear. Behaviour at maximum pump energy of 8 mJ: (c) spectral emission of the aerogel random laser as a function of pump wavelength and (d) distance between peaks (Δ*λ*) and calculated cavity length as a function of pump wavelength *λ*_Ep_.

At very low pump power, we observed the already expected linewidth narrowing and the occasional appearance of two, initially not very well defined peaks (curve 1 in Fig. 3a). However, above 300 μJ (fluence of 15 mJ mm^−2^) the two peaks stabilized and only small changes in their relative amplitude was observed from pulse to pulse. Increasing the pump power to 356 μJ maintained the appearance of the two peaks (curve 2) and increased the emission amplitude. At still higher pump powers (381 μJ) a third peak appeared (curve 3) while the emission amplitude decreased (however, the spectrally integrated power increased proportionally). No further peaks appeared at higher pump powers (461 μJ, curve 4) but, on the contrary, the relative intensity of the third peak decreased. When further increasing the pump power to 558 μJ (fluence of 28 mJ mm^−2^), there remained only one single broad peak at 559 nm and the peak at 557 nm was reduced to a small shoulder in the spectrum demonstrating the general behavior of a single peak well above threshold at gain center.

For tight focusing (*f* = 3 cm lens) the peaks appear below threshold, as shown in Fig. 3b (arrow), whereas in Fig. 2d–f (*f* = 10 cm lens) the peaks appear slightly after threshold. We attribute these effects to the reduction of the number of RL modes. Above RL threshold, a decrease of R6G absorption is expected (increase of pumping depth), which increases the ASE *Q*-factor (mainly) promoting such modes.^11,23^

A Lorentzian peak fitting was done with the spectra of Fig. 3a to determine the exact position of the peaks and their separation. The result is a gradual increase in the separation of the peaks with pump power from 1 nm to 1.4 nm, which corresponds to an equivalent linear cavity decrease with pump power from 120 μm to 86 μm. This pump power dependent cavity length decrease (as opposed to the decrease caused by a higher absorption cross-section as shown in Fig. 1e) could be caused by rhodamine desorption and rhodamine-aggregate fragmentation induced by the laser pulses.^53,54^

We further investigated this behavior of equivalent cavity length decrease with increasing gain (absorption), this time in a high gain set-up composed of high fluence (*f* = 10 cm, 8 mJ, 36 mJ mm^−2^) and large pump spot size (*w*_3_ = 260 μm). We repeated the experiments of Fig. 2d–f, changing the pump wavelength and maintaining constant fluence (shown in Fig. 3c and d). When probing the aerogel samples at high pump powers, the appearance was mostly of one single peak as is expected from the increase of *Q* factor mostly for ASE modes due to the increase of pumping depth. However, as pointed out at the beginning of Section 3, rhodamine distribution inside the aerogel matrix is expected to show large-scale inhomogeneities.^46^ Several locations on the samples, with higher absorption, showed two peaks that could be clearly attributed to one single resonator as shown by the example of Fig. 3c. These locations were used to find the equivalent cavity lengths of the resonators. No photo degradation was observed and it was possible to operate at a single location continuously for several hours or, repeatedly, switching from one location to another and back.^23^

By peak fitting Fig. 3c we calculated the separation between two peaks for all pump wavelength and calculate the respective cavity length, which is shown in Fig. 3d. Separation between both peaks resembles closely Fig. 1e because the FSR is proportional to the inverse of the cavity length, which must be proportional to the absorption length. The calculated cavity lengths in Fig. 3d range from a minimum of 11 μm to 24 μm and are much smaller than calculated from Fig. 1e. Locations in other samples that demonstrated similar coherent peaks showed approximately the same minimum cavity length (∼12 μm). As explained in Section 2, some of this enhanced local absorption feature may be attributed to the [R6G] inhomogeneity in the silica matrix that can provoke a local [R6G] increase and some may be attributed to rhodamine desorption and rhodamine-aggregate fragmentation induced by the high energy (8 mJ) laser pulses used in this set-up.^53,54^

Fig. 3c also shows that the averaged emission redshift is less than 1 nm as a function of pump wavelength, which is much less than in Fig. 2b, although absorption changes by more than a factor of seven for these pump wavelengths (see Fig. 1e). The very small red shift as a function of pump wavelength is an indication that a very high percentage (almost 100%) of the rhodamine molecules are involved in the inversion process, which could be because the R6G molecules are almost all disaggregated, increasing the sample's quantum efficiency. This is yet another indication that more rhodamine molecules must be present at these high pump powers at this specific sample locus. We note that such a small cavity (∼12 μm) reduces greatly the *Q* factor of ASE modes, which explains the absence of the ASE peak at this locus.

In Fig. 3c we observe that modes closer to the gain center receive more gain as exemplified by the emission obtained by pumping at 540 nm, which has the highest peak. Although the peaks seem much broader in Fig. 3c when compared to the narrowest peaks in Fig. 2f, the respective finesses are approximately 2.5 and 6, respectively, and therefore, the difference in Finesse is only a factor 2.4. In terms of output coupling, it means that the cavity of Fig. 2f, which works close to threshold, has losses of 65% per round-trip whereas the cavity of Fig. 3c, which works at maximum pump intensity, has almost 90% losses showing a higher gain. Part of these losses are probably due to the loss in energy needed to fragmentize and desorb the additional rhodamine molecules needed for the lasing process. These losses would be more than compensated by the much higher efficiency given the higher rhodamine efficiency and gain center concentration. In addition, a decrease of *Q* factor is also expected for such shorter cavity.

The width of the individual coherent peaks in Fig. 3c, corresponding to this cavity with ∼12 μm length, is approximately 4 nm, which corresponds roughly to the width of the ASE peak in Fig. 2a at maximum pump power (∼3.5 nm), where the cavity length is around 67 μm. This means that similar cavity *Q*-factors exist for these two specific types of peaks, being the first one a Fabry–Perot resonator and the second ASE. Clearly, a decrease of the ASE *Q*-factor is expected for the cavity with 12 μm length, which would imply in an ASE peak width several times broader than the equivalent Fabry–Perot resonator. This latter would explain the absence of the ASE background in Fig. 3c.

## 4. Conclusions

Random laser action has been studied in R6G doped silica aerogel, showing at most sites probed by the pump beam the typical single peak of amplified spontaneous emission that narrows as pumping fluence is increased above lasing threshold. Below RL threshold, several peaks with a regular frequency spacing are observed as pumping energy is increased. The peak frequencies and their spacings was associated to the formation of effective resonators (Fabry–perot) in the pumping region (depth). The number of modes of a single resonator goes from one to many modes, which then decreases again to one single peak for higher pumping energies. The typical behaviour of a classical random laser is observed, which is many modes at threshold and, with increasing pump power and gain competition, one single ASE mode. At specific sample sites and very high pump powers, it is also possible to observe two peaks belonging to one set of modes, which was associated to a strong decrease of pumping depth due to an increase in effective absorption. This latter was attributed to a higher [R6G] (inhomogeneity) inside the aerogel, which would be desorbed from the silica surface and disaggregated by the high energy.

## Author contribution

N. U. W. & A. R. M. performed RL experiments; E. P. & S. J. L. R. Synthesized silica aerogel; N. U. W. & E. J. V. analyzed the results and wrote the manuscript.

## Conflicts of interest

There are no conflicts to declare.

## Supplementary Material
