# Correction: Dynamic random lasing in silica aerogel doped with rhodamine 6G

**DOI:** 10.1039/c8ra90081g

**Published:** 2018-10-22

**Authors:** Niklaus Ursus Wetter, Adriana Ramos de Miranda, Édison Pecoraro, Sidney José Lima Ribeiro, Ernesto Jimenez-Villar

**Affiliations:** Centro de Lasers e Aplicações, CNEN-IPEN/SP Av. Prof. Lineu Prestes 2242 São Paulo CEP 05508-000 Brazil nuwetter@ipen.br; Instituto de Química de Araraquara, Universidade Estadual Paulista Júlio de Mesquita Filho Rua Prof. Francisco Degni 55, Araraquara São Paulo CEP 14800-900 Brazil

## Abstract

Correction for ‘Dynamic random lasing in silica aerogel doped with rhodamine 6G’ by Niklaus Ursus Wetter *et al.*, *RSC Adv.*, 2018, **8**, 29678–29685.

The authors regret that there was an error in ref. 8 in the original article. The correct reference should read: E. Jimenez-Villar, M. C. S. Xavier, N. U. Wetter, V. Mestre, W. S. Martins, G. F. Basso, V. A. Ermakov, F. C. Marques and G. F. de Sá, *Photonics Res*., 2018, **6**, 929–942, DOI: 10.1364/prj.6.000929.

The Royal Society of Chemistry apologises for these errors and any consequent inconvenience to authors and readers.

## Supplementary Material

